# Determination of Sex from Footprint Dimensions in a Ghanaian Population

**DOI:** 10.1371/journal.pone.0139891

**Published:** 2015-10-07

**Authors:** Jubilant Kwame Abledu, Godfred Kwame Abledu, Eric Bekoe Offei, Emmanuel Mensah Antwi

**Affiliations:** 1 School of Veterinary Medicine, College of Basic and Applied Sciences, University of Ghana, Accra, Ghana; 2 Department of Applied Mathematics, Faculty of Applied Sciences and Technology, Koforidua Polytechnic, Koforidua, Ghana; West Virginia University, UNITED STATES

## Abstract

The present study sought to verify the utility and reliability of footprint dimensions in sex determination in a Ghanaian population. Bilateral footprints were obtained from 126 Ghanaian students (66 males and 60 females) aged 18–30 years at Koforidua Polytechnic using an ink pad and white papers. Seven dimensions–length of each toe (designated T1-T5) from the most anterior point of the toe to the mid-rear heel point, breadth at ball (BAB) and breadth at heel (BAH)–and the heel-ball (HB) index were obtained from each footprint. Some footprint dimensions (i.e. T2, T3, T4 and T5) showed statistically significant bilateral asymmetry in males only. All the footprint dimensions, except HB index, were significantly greater in males than females (p<0.001). Applied singly in discriminant function analysis, the footprint dimensions allowed 69.8%-80.3% of cases to be correctly classified into their sex groups; the accuracy of sex classification was higher using left footprints than right footprints. With all dimensions subjected to stepwise discriminant function analysis 80.3% and 77% of cases could be correctly classified, combining both T5 and BAH for left footprints and T1, BAB and BAH for left footprints respectively. The present study has demonstrated, for the first time among Ghanaian subjects, the utility and reliability of sex determination standards developed from footprint dimensions. The results thus provide the baseline for elaborated studies in the future.

## Introduction

The human foot is a highly complex structure consisting of 26 major bones and numerous synovial joints [1]. It plays a role in both load support and shock absorption as well as providing balance and stabilization of the body during gait [1,2]. The morphology of human foot varies considerably due to the combined effects of heredity, lifestyle, and climatic factors [3]. In addition, natural biological variance, age, population group, BMI, parity and sex have significant influences on the morphology of an individual’s foot [4].

Sex differences in foot morphology have important applications in footwear design [4,5] and forensic anthropology [6–8]. Wunderlich and Cavanagh [5] showed that men had longer and broader feet than women for any given stature. In addition, male feet differs from female feet in a number of shape characteristics, particularly at the arch, the lateral side of the foot, the hallux and the ball of the foot [5]. Anthropometric studies have recorded significantly larger values for various dimensions of the foot in males than in females [4–8]. Studies have shown that contact area at any region of the plantar surface of the foot is greater in men than in women [9,10]. Sex differences in foot morphology and contact area imply sex differences in plantar pressure distribution, although empirical results are inconsistent [9,10].

A footprint is an impression of the weight-bearing areas of the plantar surface of the foot. Footprints can be found on rain covered surfaces, newly waxed floors, freshly cemented surfaces, moistened surfaces, in dust, mud, sand, oil, paint and blood at murder scenes [11,12]. Footprints can be found at crime scenes because offenders often remove their footwear, either to avoid noise or to gain a better grip in climbing walls, etc, while entering or exiting [13]. Like fingerprints, footprints of an individual are unique to that individual [14–17]. Hence, footprints linked to a crime can be compared with a suspect’s footprints as a means of confirming or ruling out involvement in that crime.

The shape of a footprint is influenced by a complex of anatomical, functional, and sedimentary (surface) variables [18]. The depth of a footprint varies with plantar pressure distribution depending on the nature and type of the substrate [18,19]. Therefore, sex differences in plantar contact area and plantar pressure distribution imply sex differences in footprint morphology. Several studies support the existence of dimensional sexual dimorphism in footprint morphology [6,20,21]. Other studies suggest strong links between footprint dimensions and stature [3,11,13,22–24], body weight [12,23,25] and holding weight [25]. Thus, analysis of footprints can help in the determination of sex and estimation of stature, body weight and holding weight of an individual in forensic investigations.

The determination of sex is one of the first and most important steps in establishing personal identity in forensic investigations. The most popular statistical model for sex determination in forensic investigations is the discriminant function analysis [26,27]. The discriminant functions developed from footprint data for a particular population cannot be applied universally since people from different populations differ in their foot morphology; population-specific standards are thus necessary for improved sex determination.

Presently in Ghana, there is a lack of forensic databases for sex determination from footprints. This preliminary study, therefore, sought to verify the utility and reliability of footprint dimensions in sex determination, and establish population-specific discriminant functions for sex determination in a Ghanaian population.

## Materials and Methods

### Study subjects

The study was carried out among 126 Ghanaian students (66 males and 60 females) aged 18–30 years and of different ethnic and socio-demographic backgrounds at Koforidua Polytechnic, Koforidua in the eastern region Ghana. The study participants were healthy and free from any apparent symptomatic deformity of the foot. Participation in the study was voluntary and entirely based on written informed consents. The consent forms were signed and returned by all the participants. The study protocol, including the consent procedure was reviewed and approved by the Committee on Human Research, Publication and Ethics of the School of Medical Science, Kwame Nkrumah University of Science and Technology and the Komfo Anokye Teaching Hospital, Kumasi, Ghana.

### Data collection and anthropometry

#### Footprints

**Fig 1:**
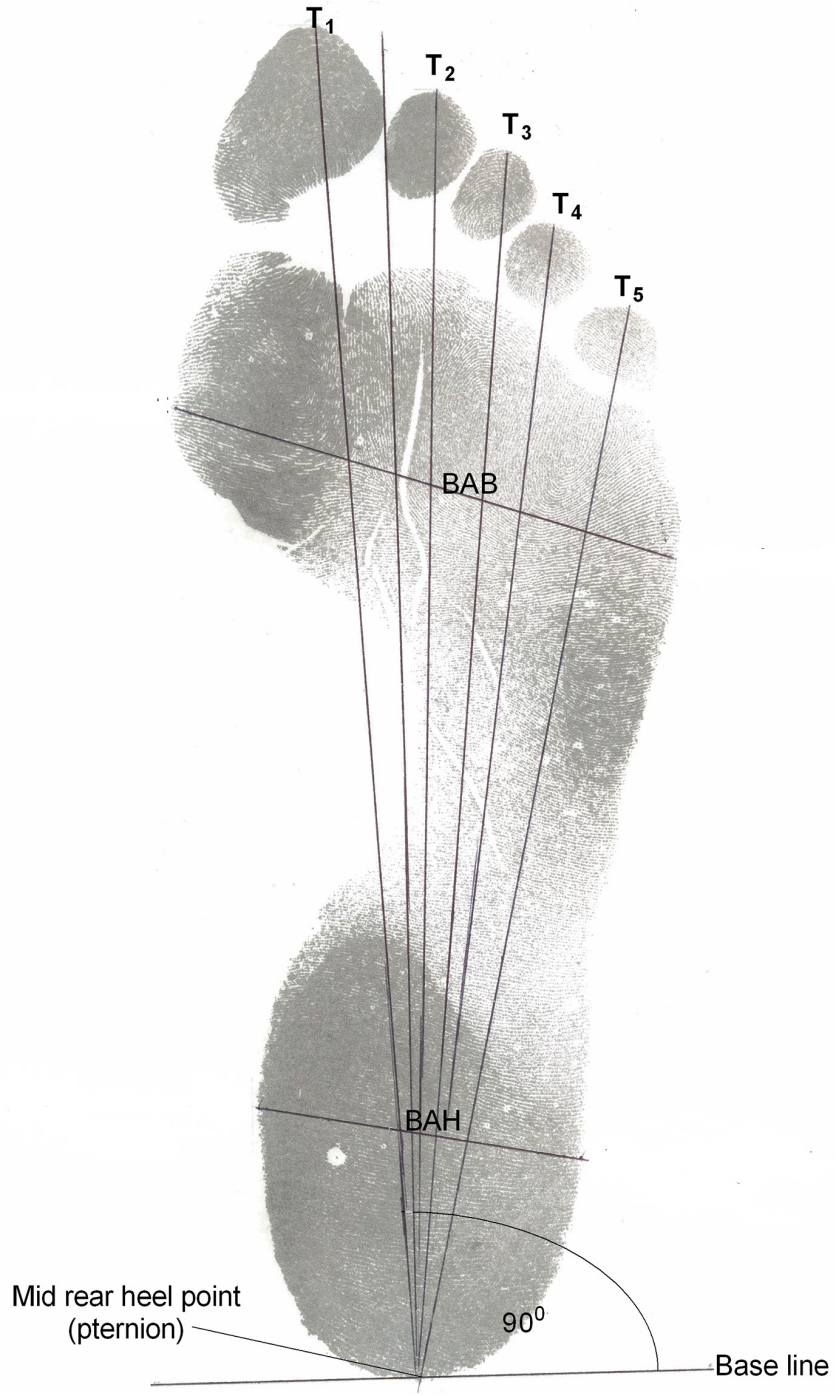
Footprints landmarks and dimensions.

Two hundred and fifty-two bilateral footprints were obtained from the study participants using an inkpad, with a non-reactive, non-indelible black ink. After cleansing their feet, the participants were requested to step their soles on the inkpad with minimal pressure, and then transfer the inked foot onto a plain white paper kept aside on a flat surface. Left and right footprints were recorded one by one for each participant. A total of 7 measurements, comprising five length dimensions and two breadth dimensions (as described by Hemy et al [6]) were obtained from left and right footprints of each participant using a measuring rule (The Perfect Measuring Tape Company, USA). To establish a definite axial orientation for measurement, two important landmarks–the designated longitudinal axis (DLA) and base line (BL)–were marked on the footprints following procedures described by Krishan [11]. The DLA was drawn as a straight line from the pternion (i.e the most posterior point of the rear heel margin) to the lateral side of the first toe pad margin. Base line (BL) was drawn perpendicular to the DLA at the rear edge of the footprint, extending from the pternion in both medial and lateral directions. The following measurements were taken on each footprint (Fig 1):

- T1- Length measurement taken from the pternion (P) to the most anterior point of toe 1.
- T2- Length measurement taken from the pternion (P) to the most anterior point of toe 2.
- T3- Length measurement taken from the pternion (P) to the most anterior point of toe 3.
- T4- Length measurement taken from the pternion (P) to the most anterior point of toe 4.
- T5- Length measurement taken from the pternion (P) to the most anterior point of toe 5.
- Breadth at ball (BAB) - Measurement between the most lateral and the most medial projecting points of the footprint margin at the ball (which corresponds to the most prominent areas of the metatarsal-phalangeal joints).
- Breadth at heel (BAH) - Measured as the widest distance across the heel.

An additional variable, heel-ball (HB) index was calculated as (BHEL ÷ BBAL) × 100 for all the footprints. To avoid inter-observer error, the measurements were performed by one observer and recorded to the nearest 0.1 centimeter. Before data collection, all measurements were taken twice on 15 subjects selected randomly from the sample. The means of these two measurements were then compared statistically using paired t test; a non-significant result (p>0.05) indicated that the two measurements were identical and reproducible without significant intra-observer errors.

### Statistical analysis

Footprint measurements were compared for bilateral and sex differences using paired and unpaired t tests respectively. Sexually dimorphic measurements were subjected to univariate and stepwise multivariate discriminant analyses. The discriminant function (D) for the determination of sex from footprint measurements is given as:

$$D=b_{0}+\sum_{i}b_{i}X_{i}$$

Where b_0_ and b_i_ represent the coefficients of the discriminant function and X_i_ represents the footprint measurement. The discriminant functions were evaluated considering the Wilks’ lambda, eigenvalue and canonical correlation. The decision rule for sex discrimination was based on sectioning points (S) derived for each discriminant function. An individual was classified as male if the value of the discriminant function (D) was greater than S. If the value of D was lesser than S, the individual was classified as female. Data analysis was performed by using Statistical Package for Social Sciences (SPSS) version 20 for Windows (IBM Company, Chicago, IL). All statistical analyses were considered significant if p<0.05.

## Results

The means, standard deviations and differences between left and right footprint dimensions in both males and females are shown in Table 1. In females, no statistically significant differences were observed between the left and right footprint dimensions (p>0.05). In males, however, all dimensions except T_1_, BAB and BAH were significantly greater in the left footprints than right footprints (p<0.001).

**Table 1:** Means, standard deviation and left-right differences of footprint measurements stratified by sex.

		*Male*			*Female*	
	*Right*	*Left*	*paired*	*Right*	*Left*	*paired*
*Parameter*	*(n = 66)*	*(n = 66)*	*t-test*	*(n = 60)*	*(n = 60)*	*t-test*
T_1_	24.9±1.2	25.1±1.2	1.990	23.5±1.2	23.5±1.0	0.942
T_2_	24.7±1.3	24.9±1.3	3.495*	23.1±1.2	23.2±1.1	0.199
T_3_	23.8±1.2	23.9±1.2	3.393*	22.3±1.2	22.3±1.1	0.667
T_4_	22.6±1.1	22.7±1.0	3.794*	21.2±1.1	21.2±1.0	0.668
T_5_	21.1±1.1	21.2±1.0	3.521*	19.5±1.3^β^	19.7±0.8¥	0.154
BAB	9.6±0.5	9.6±0.5	0.655	8.9±0.6	8.9±0.7	0.565
BAH	5.7±0.5	5.7±0.5	1.443	5.1±0.5	5.0±0.5	0.306
HB index	58.7±5.1	59.0±5.5	0.784	57.0±5.3	57.0±6.3	0.597

Data are presented as mean ± standard deviation. T_1:_ length from anterior part of 1st toe to mid-rear heel point; T_2_: length from anterior part of 2nd toe to mid-rear heel point. T_3_: length from anterior part of 3rd toe to mid-rear heel point; T_4_: length from anterior part of 4th toe to mid-rear heel point; T_5_: length from anterior part of 5th toe to mid-rear heel point. BAB: breadth at ball; BAH: breadth at heel; HB index: heel-ball index;

β: n = 59 and

¥: n = 56 due to missing toes.

*p<0.001 when left and right values were compared.

Table 2 shows the differences between male and female footprint dimensions. Apart from the heel-ball (HB) index, all the footprint dimensions were statistically greater in males than females (p<0.001).

**Table 2:** t test for sexual differences in left and right footprint measurements.

	Footprint parameter							
	T_1_	T_2_	T_3_	T_4_	T_5_	BAB	BAH	BH Index
***Right***	7.976*	7.840*	8.126*	8.578*	9.322*	7.422*	6.886*	1.878^¥^
***Left***	6.768*	6.519*	6.410*	6.493*	6.181*	7.265*	6.019*	1.719^¥^

Data are presented as mean ± standard deviation. T_1:_ length from anterior part of 1st toe to mid-rear heel point; T_2_: length from anterior part of 2nd toe to mid-rear heel point. T_3_: length from anterior part of 3rd toe to mid-rear heel point; T_4_: length from anterior part of 4th toe to mid-rear heel point; T_5_: length from anterior part of 5th toe to mid-rear heel point. BAB: breadth at ball; BAH: breadth at heel; HB index: heel-ball index.

* p<0.001 and

¥ p˂0.05 when male and female values were compared.

The results of the univariate discriminant function analysis are presented in Table 3. For left and right footprints respectively, the discriminant functions with the highest eigenvalues and lowest Wilks’ lambda values were produced by T_5_ and BAB. On the other hand, the discriminant functions with the least eigenvalues and highest Wilks’ lambda values were produced by BAH for both left and right footprints. Univariate discriminant function analysis showed that 71.4%–80.3% and 69.8%-76.2% of cases were correctly into their sex groups using left and right footprint dimensions. T_5_ (of left footprints) and BAB (of right footprints) yielded the most accurate discriminant functions while BAH (of left footprints) and T_3_ (of right footprints) yielded the least accurate discriminant functions. Furthermore, the discriminant functions showed biasedness in terms of sex classification. T_1_, T_2_, T_3_, T_5_ and BAH of left footprints and T_2_ of right footprints were better in classifying females than males. T_4_ and BAB of left footprints and T_1_, T_3,_ T_4_, T_5_, BAB and BAH of right footprints were better in classifying males than females. Stepwise discriminant function analysis of all the footprint dimensions retained both T5 and BAH for left footprints and T1, BAB and BAH for right footprints. The stepwise analysis correctly classified into their sex groups 80.3% and 77% of the cases using left and right footprints respectively (Table 4).

**Table 3:** Univariate discriminant function analysis of footprint dimensions.

								Classification of correct group membership		
Function	Parameter	*b*_*0*_	*b*_*1*_	C	Wilks' lambda	Eigen-value	*S*	Male (n = 66) n (%)	Female (n = 60) n (%)	Total (n = 126) n (%)
***Left footprints***										
1	T_1_	-21.348	0.878	0.582	0.661	0.513	-0.068	48(72.7)	48(80.0)	96(76.2)
2	T_2_	-20.002	0.831	0.576	0.669	0.496	-0.067	46(64.7)	48(80.0)	94(74.6)
3	T_3_	-20.145	0.870	0.587	0.653	0.533	-0.069	48(72.7)	45(75.0)	93(73.8)
4	T_4_	-21.539	0.979	0.610	0.628	0.593	-0.073	52(78.8)	46(76.7)	98(77.8)
5	T_5_	-22.243	1.084	0.648	0.580	0.724	-0.139	53(80.3)	45(80.4)^¥^	98(80.3)^≠^
6	BAB	-15.950	1.716	0.555	0.692	0.444	-0.063	54(81.8)	44(73.5)	98(77.8)
7	BAH	-10.248	1.904	0.524	0.725	0.379	-0.055	46(69.7)	44(73.3)	90(71.4)
***Right footprints***										
8	T_1_	-20.050	0.828	0.519	0.730	0.369	-0.057	49(74.2)	42(70.0)	91(72.2)
9	T_2_	-18.678	0.782	0.505	0.745	0.343	-0.055	45(68.2)	45(75.0)	90(71.4)
10	T_3_	-18.845	0.818	0.499	0.751	0.331	-0.045	49(74.2)	39(65.0)	88(69.8)
11	T_4_	-19.566	0.894	0.504	0.746	0.340	-0.055	47(71.2)	42(70.0)	89(70.6)
12	T_5_	-15.934	0.786	0.487	0.763	0.311	-0.062	50(75.8)	38(64.4)^α^	88(70.4)^β^
13	BAB	-17.464	1.878	0.546	0.701	0.426	-0.062	53(80.3)	43(71.7)	96(76.2)
14	BAH	-10.728	1.998	0.476	0.774	0.292	-0.051	48(72.7)	41(68.3)	89(70.6)

T_1:_ length from anterior part of 1st toe to mid-rear heel point; T_2_: length from anterior part of 2nd toe to mid-rear heel point. T_3_: length from anterior part of 3rd toe to mid-rear heel point; T_4_: length from anterior part of 4th toe to mid-rear heel point; T_5_: length from anterior part of 5th toe to mid-rear heel point. BAB: breadth at ball; BAH: breadth at heel. b_0_, b_1_ –constants of discriminant function, S-sectioning point, C- canonical correlation coefficient.

¥: n = 56

≠: n = 122

α: n = 59 and

β: n = 125 due to missing toes.

**Table 4:** Stepwise multivariate discriminant function analysis of footprint dimensions.

										Classification of correct group membership		
Function	Parameter	b_o_	b_1_	b_2_	b_3_	C	Wilks' lambda	Eigen-value	S	Male (n = 66)n (%)	Female (n = 60) n (%)	Total (n = 126) n (%)
***Left footprints***												
15	T_5_ + BAH	-21.764	0.866	0.741	-	0.676	0.543	0.841	-0.149	51(77.3)	47(83.9)^¥^	98(80.3)^≠^
***Right footprints***												
16	T_1_ + BAB + BAH	-20.406	0.350	0.862	0.730	0.606	0.633	0.579	-0.084	52(78.8)	45(75.5)	97(77.0)

T_1:_ length from anterior part of 1st toe to mid-rear heel point; BAB: breadth at ball. b_0_, b_1_ –constants of discriminant function, S-sectioning point, C- canonical correlation coefficient.

¥: n = 56 and

≠: n = 122 due to missing toes.

## Discussion

The footprint dimensions in this study can be compared with those reported by Ukoha [3] among Nigerians with similar socio-cultural characteristics. The footprint dimensions of the subjects in the present study exhibit slightly lower values compared with the Nigerian counterparts. This inter-population variation of footprint dimensions indicates the need for the establishment of population-specific standards for improved forensic identification.

Furthermore, the results indicated that some footprint dimensions (i.e. T_2_, T_3_, T_4_ and T_5_) showed statistically significant bilateral asymmetry; these dimensions were greater in left footprints than right footprints, and present only in males. Populations studies have reported the existence of bilateral asymmetry in different footprint dimensions [3,11,22], suggesting that left and right feet of the same individual may not make identical footprints. While the observed bilateral asymmetry may be attributed to the ‘dominant foot’ phenomenon postulated by previous researchers [11,22], its occurrence exclusively in males could not readily be inferred from this study. Conversely, a study by Hemy et al [6] found no significant bilateral asymmetry in footprint dimensions of both males and females.

As expected, all the footprint dimensions were significantly greater in males than females (Table 2). This finding is consistent with the general agreement that men have longer and broader feet than women [4,5]. Wunderlich and Cavanagh [5] demonstrate that female feet are not merely scaled-down versions of male feet but also have a higher arch, a shallower first toe, a shorter ankle length, a shorter length of the outside ball of foot, and a smaller instep circumference than men with similar foot length. The fact that footprint dimensions are sexually dimorphic is supported by several researchers [6,21,28].

Discriminant function analysis indicated that an individual’s sex could be predicted from their footprint dimensions (Table 3). The accuracy of the discriminant functions varied from 69.8% to 80.3%, and was better using left footprints (i.e. 71.4%–80.3%) than right footprints (i.e. 69.8%-76.2%). Recent studies among Western Australians [6] and Turks [21] showed that using discriminant function analysis, 79.5%–89.5% and 66.7%-82.4% of individuals could be respectively classified using their footprint dimensions. The footprint dimension that wielded the highest accuracy of sex discrimination varied from the longest toe [6] to the third toe (T_3_) [21] in different populations. In the present study, the most accurate discriminant functions were produced by T_5_ and BAH using left and right footprints respectively.

Stepwise multivariate discriminant function analysis selected both T_5_ and BAH of left footprints and T_1_, BAB and BAH of right footprints as the best combination of footprint dimensions for optimum sex discrimination. The stepwise analysis correctly classified 80.3% and 77% of cases into their sex groups using left and right footprints respectively. This finding thus agrees with earlier studies [6,21], which indicated that even if all footprint dimensions were jointly used, a perfect (i.e 100%) accuracy of sex determination would be unattainable. Nonetheless, the accuracy and reliability of the discriminant functions were by and large, good for sex determination in this Ghanaian population. The discriminant functions can be used in conjunction with individualizing characteristics of footprints [14,16,29] to achieve a perfect or near-perfect accuracy of sex determination during forensic investigations.

The present study is limited by its relatively small sample size (n = 126), thus the results cannot be generalized. Nonetheless, these preliminary results provide the baseline for elaborated studies in the future.

## Conclusion

The current study has demonstrated, for the first time among Ghanaian subjects, the utility and reliability of sex determination standards developed from footprint dimensions. Using discriminant function analysis, the current study has shown that the accuracy of footprint dimensions in sex determination is high (i.e 69.8%-80.3%) and better using left footprints than right footprints. These findings have important applications in personal identification during forensic investigations. Further studies involving large samples of different age and ethnic groups in Ghana could enhance the forensic relevance of the present results.

## Supporting Information

S1 File Footprint dimensions. (ZIP)
